# Effects of Visual Context upon Functional Connectivity during Observation of Biological Motions

**DOI:** 10.1371/journal.pone.0025903

**Published:** 2011-10-04

**Authors:** Magaly Hars, Mélany Hars, Cornelis J. Stam, Claire Calmels

**Affiliations:** 1 de l'Expertise et de la Performance, INSEP, Institut National du Sport, Paris, France; 2 Université de Reims Champagne-Ardenne, Reims, France; 3 Department of Rehabilitation and Geriatrics, Geneva University Hospitals, Geneva, Switzerland; 4 LAMIH UMR CNRS 8530, Université de Valenciennes et du Hainaut Cambrésis, Valenciennes, France; 5 Department of Clinical Neurophysiology, VU University Medical Centre, Amsterdam, The Netherlands; The University of Western Ontario, Canada

## Abstract

The aim of this study was to examine brain responses, in particular functional connectivity, to different visual stimuli depicting familiar biological motions. Ten subjects actively observed familiar biological motions embedded in point-light and video displays. Electroencephalograms were recorded from 64 electrodes. Activity was considered in three frequency bands (4–8 Hz, 8–10 Hz, and 10–13 Hz) using a non-linear measure of functional connectivity. In the 4–8 Hz and 8–10 Hz frequency bands, functional connectivity for the SMA was greater during the observation of biological motions presented in a point-light display compared to the observation of motions presented in a video display. The reverse was observed for the 4–8 Hz frequency band for the left temporal area. Explanations related to: (i) the task demands (i.e., attention and mental effort), (ii) the role(s) of theta and alpha oscillations in cognitive processes, and (iii) the function(s) of cortical areas are discussed. It has been suggested that attention was required to process human biological motions under unfamiliar viewing conditions such as point-light display.

## Introduction

It is recognized that the human visual system possesses specific and impressive properties: Individuals are able to identify biological motions from impoverished contexts such as point-light displays [1] in which a few key points of light are mounted on a moving human body. Individuals are even able to recognize the gender of a person or the emotions experienced by this person from these impoverished displays (see [2] for a review). However, this ability to recognize biological motions presents some limits. It is disrupted when the point-light display is inverted or watched under dim light conditions (see [2] for a review). This observation would demonstrate the significant role of the contextual information (i.e., form, shape, colour, texture, overt muscle contraction) in the perception of biological motions.

At present, there are two points of view on the mechanisms involved in the perception of human motion. On one hand, processing of human/biological motion requires bottom-up processing [1]–[4]. Motion processing is viewed as an automatic and effortless function. On the other hand, the second point of view stresses the participation of top-down mechanisms such as attention [5]–[11]. Studies have shown that attention was required to analyze human biological motions under degraded, ambiguous viewing conditions and when competing stimuli were presented.

Considering the two theoretical points of view mentioned previously, it would be of interest to investigate whether the active observation of a highly familiar biological motion embedded in an unusual display, never encountered before, involves the same processing at play during the observation of the same familiar motion embedded in a familiar display. Viewing a mundane motion within a familiar display requires a bottom-up processing [2]–[4] whereas watching this motion within an unfamiliar display may require, in addition to the bottom-up processing, a top-down processing.

A point-light display can be considered as an unfamiliar impoverished display never experienced in every day life by individuals since it is an artificial display generated by a complex technology (i.e., optoelectronic Vicon 612 system) where contextual information were withdrawn. Conversely, a familiar display is a “normal”, ecological display laden with contextual information. In the brain-imaging literature, two studies [12], [13] have compared the cortical responses obtained when subjects observed point-light and “normal” video displays. Beauchamp et al. [12] demonstrated that the superior temporal sulcus (STS) was more strongly activated by video displays than point-light displays whereas the reverse was observed within the middle temporal gyrus (MTG). In contrast, Grossman and Blake [13] did not observe any BOLD response differences in the posterior STS between these two displays. In both studies, biological motions included walk, jumping jack, kick, and thrown.

To shed some light on the processing involved in human visual perception of biological motions, the present study aimed to study the cortical mechanisms enrolled in response to different visual stimuli depicting highly familiar biological motions. Highly familiar motions were embedded in a highly familiar display (i.e., “normal” display as if you watch movies on the television) and a highly unfamiliar impoverished display (i.e., point-light display). Subjects were chosen among a population of expert female gymnasts who possessed a visual and motor familiarity with the motions they were going to observe. This recruitment procedure ensured that the subjects were motorically and visually highly accustomed to the stimuli employed in the present study and that the factor which had been manipulated was the display (unfamiliar *vs* familiar). Cerebral rhythmic activities over the scalp during the observation of biological motions within point-light and video displays were compared. Motion observation was completed with the purpose of later recognition. Cerebral activities were assessed using the synchronization likelihood (SL) measure which is an EEG indicator of linear and non-linear changes in functional connectivity between different brain areas [14], [15]. Using SL measure compared to more conventional power or coherence analyses is of prime importance since SL measure demonstrates some advantages. For instance, in contrast to event-related desynchronization [16]–[18], which is an indicator of power change argued as revealing only part of the relevant information since it can only be used as an index of local cortical engagement [19], the SL measure allows the detection of interactions between brain regions. SL measure also involves an ability to characterize non-stationary data with rapidly changing interdependencies and identification of non-linear interdependencies between the underlying dynamical system (see [15], [19], [20] for further details of these concerns) which are important considerations for EEG research [21]. This was not the case of traditional measures of coherence, which estimate the similarity between time series of electrical potential via linear techniques.

Motor related areas, temporal and occipito-parietal areas were considered since these are known to play a role in action observation [22]–[28]. More specifically, the selection of brain areas, in the present study, has been based on Gazzola and Keyser's model [28]. Their proposal states that during observation, an inverse model involving the middle temporal gyrus (MTG), the posterior parietal cortex (PPP), the premotor cortex (PM), the primary motor cortex (M1), the primary somatosensory cortex (SI), and the cerebellum is operating. During action observation, MTG sends visual information into the PPC which in turn transmits it to SI and PM through a cortico-cortical pathway, and to the cerebellum and PM through a cortico-cerebellar-cortical pathway. When the information attains the PM, mesial wall areas (e.g., SMA) seem to forbid premotor activity to access M1 to prevent the development of overt actions.

We hypothesized that during the observation of a familiar biological motion, processing involved under a point-light observation condition would be different to those involved under a video observation condition. Functional connectivity under a point-light observation condition is expected to be greater than under a video observation condition. Extra processing like top-down mechanism such as attention may be required to process and interpret point-light displays since individuals are unaccustomed to experiencing these artificial stimuli in the daily life. This would entail more effort as it has been perceived as a difficult task and may be expressed by a higher functional connectivity. Differences in functional connectivity were also conjectured to occur in lower frequency bands. Oscillations in theta band are recognized as being related to encoding processes [29] and mental effort [30], [31]. In contrast, oscillations in the lower alpha frequency band are known to be associated to attentional processes [30].

## Materials and Methods

### Subjects

Fifteen French national female gymnasts, who had normal vision and no past neurological or psychiatric history, participated voluntarily in the study. The subjects were uninjured at the time of the study and were not informed of the goals of the study. Five subjects were discarded from the study due to electrode impedance values superior to 5 kΩ and to noisy EEG waveforms. Data from the ten remainder subjects (mean age = 20.9, SD = 3.14) were considered for further analysis.

Before inclusion, each gymnast completed a questionnaire to assess their visual and motor familiarity with the acrobatic movements shown in the experiment. From a practical point of view, each acrobatic movement has been designed by its well-established name in gymnastics terminology and the gymnasts had to answer the following two questions: (i) How often do you see this movement? (Visual familiarity) and (ii) How often do you perform this movement? (Motor familiarity). A 10-point Likert-type scale was used for scores. The scale was structured accordingly: “0” never and “10” very often. Gymnasts, whose scores were below “8”, were discarded from the study. This procedure suggests that the selected subjects possessed a strong visual and motor familiarity with the movements employed in the experimental procedure. They perceived them as being both usual and familiar. Though male national gymnasts were visually highly accustomed to the movements performed by their female counterparts, they were not recruited since they did not possess a motor familiarity with these movements. In gymnastics, most of the movements were specific to one sex and had therefore not been physically practiced by the other gendered group. Including a male sample in the present study would have led to bias. Indeed, when viewing a biological motion, the activation within the parietal, premotor areas, and the superior temporal sulcus, which were our areas of interest, was modulated by the motor familiarity/motor competence of the observer. When the observed movements do not belong to the observer's motor repertoire, only limited activation is seen within these areas in contrast to the activation revealed when movements are physically mastered by the observer [32], [33].

Finally, all the subjects were assessed as strongly right handed by the Edinburgh Handedness Inventory (EHI score = 92.9/100) [34]. They gave written informed consent and separate parental consents were also obtained for the subjects who were under the age of 18. The study was conducted in accordance with the Declaration of Helsinki and approved by the local ethics committee (Comité de Protection des Personnes d'Ile de France VI, CPP, and Agence Française de Sécurité Sanitaire des Produits de Santé, AFSSAPS, ID RCB: 2009- A00934-53).

### Task and Production of Videos

An international female gymnast, who did not participate in the present study, performed 30 series of four acrobatic movements. These series were matched for difficulty. They were selected among a panel of 80 possible connections of four acrobatic movements performed by the international female gymnast. Two national standard judges were invited to assess the difficulty to remember these series on a 5 point-Likert scale after observing these series in a live condition. Connections assessed as easy to remember (i.e., assessed 1, 2, or 3 on a 5 point-Likert scale) were discarded from the study. The series which were retained were composed of three different acrobatic movements with movements that resembled each other.

The international female gymnast was filmed in a gymnasium on the floor area whilst she performed the 30 connections. In the first case, a digital camera was used to obtain 30 ten second-colour videos. In the second case, the same series were registered via the optoelectronic Vicon 612 system to generate point-light displays lasting 10 sec. Eight infrared cameras (Charge Coupled Deviced) sampling at 120 Hz registered the spatiotemporal positions of 32 retroreflective markers. These were located at the conventional standardized marker set (Plug-In-Gait markers, Vicon Motion Systems). Two kinds of stimuli were obtained. The first stimuli took into account the colour, the shape, the shading, and the contours of the acrobatic movements (i.e., video motion) whereas the second one was much simplified and characterized the movements by dots of light (i.e., point-light motion). The point-lights were displayed in a white against a black background.

### Experimental Procedure

The subjects were invited to complete four conditions: (i) two control conditions, (ii) a video motion observation condition, and (iii) a point-light motion observation condition. During these conditions, EEG was recorded. After the acquisition of EEG data, the subjects were invited to assess, via a 10-point Likert scale (“0” very difficult and “10” very easy), the difficulty to observe and recognize movements under a video and a point-light motion observation conditions.

#### Control conditions

The two control conditions were systematically presented first. Subjects were just informed to passively observe, in the first control condition, a static shot of the area in which the international gymnast had performed the 30 connections and in the second control condition, a black screen. Each control condition lasted one minute. The first condition was used as a control for the video motion observation condition, whereas the second one served as a control for the point-light motion observation condition. The observation of a background without a static agent stimulus was adopted based on previous studies. Grafton and co-workers [35] reported that observing a movement was better contrasted with the observation of an inert object or an ‘empty’ background than the observation of a static hand. Recently, Jonas et al. [36] and Urgesi et al. [37] have suggested that viewing a stationary hand (suggesting a transition to action) was sufficient for activating motor related areas. Similarly, work by Grossman et al. [38] and Saygin et al. [39] has shown that scrambled biological motions recruited, to a lesser extent, areas which were responsive to the view of biological motions.

#### Video motion observation condition

**Figure 1:**
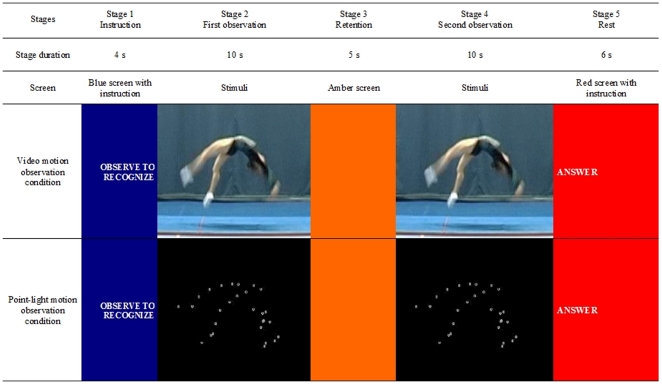
Schema for one trial according to observation conditions.

The video motion observation condition was composed of 30 trials. Each trial, which lasted 35 s, comprised five stages which were shown via a video display. Different screen colours (blue, amber, and red) helped the subject follow the procedure (see Figure 1). In stage 1 (blue screen) lasting 4 s, the subject received an instruction to observe the series of acrobatic movements with the aim of recognizing it subsequently. In stage 2, lasting 10 s, the subject viewed the video motion of the series of acrobatic movements. After the video motion observation, the subject was asked to remain focused for 5 s (stage 3, amber screen) before completing the recognition task. In stage 4, lasting 10 s, a second video motion was presented and the subject had to decide whether this video was similar or dissimilar to that viewed in stage 2. Altogether, 50% of the videos were similar. Clenching or not clenching the fist was used to indicate the response in stage 5 (red screen). In this last stage, the subject was encouraged to relax and to blink their eyes if necessary.

#### Point-light motion observation condition

In the point-light motion observation condition, the 30 trials were conducted in a similar way to those used in the video motion observation condition. However, stages 2 and 4 were different. Though the subjects observed the same set of series of acrobatic movements that were displayed in the video motion observation condition, the display under the point-light motion observation condition was impoverished and characterized by dots of light (see Figure 1).

Synchronization between the EEG signal and the videos was carried out using a photoresistive diode which responded to the screen colour change. The two control conditions were presented first to the subjects. Then, the subjects were provided with the instructions of the experiment. This was followed by a 10 minute-period in which the subjects had an opportunity to familiarize themselves with the tasks they had to perform. After the training session, the experiment itself began. The 60 trials (i.e., 30 trials for the video motion observation condition and the 30 trials for the point-light motion observation condition) were randomized and distributed at random among four 8 min 45 s blocks. Each block was thus composed of 15 trials stemming from the two observation conditions. A five minute rest period separated each block. The experimenter monitored the correctness of the answer provided in the two observation conditions for recognition. Incorrect answers were discarded from further analysis.

### Data Acquisition and Recording

Electrical brain activity was recorded from 64 electrodes mounted in an elastic lycra cap (SynAmps2 64 channels Quick-Cap, Neuromedical supplies, Charlotte, NC, USA) and placed in accordance with the international 10-10 system [40]. Mastoids were used for the reference electrodes and the ground electrode was located between FPZ and FZ. Electro-oculograms (EOG) were also registered from the canthi of both eyes (horizontal EOG) and the supra and infra orbital of the left eye (vertical EOG). Electrode impedance was kept homogenously below 5 kΩ throughout the experimentation. Amplifier bandwidth was set between 0.05 and 100 Hz. Initial sampling frequency was 500 Hz. For further analysis data were downsampled to 250 Hz. AD resolution was 24 bit.

### Synchronization Likelihood

Synchronization Likelihood (SL) is a general measure of linear and non-linear correlations between EEG signals [14], [15] which can be used on short epoch lengths [41]. This measure characterizes interchannel synchronization and is the likelihood that recurrence of a pattern in time series X at two times i and j will coincide with recurrence of patterns in time series Y at the same times i and j. The patterns are defined in terms of state-space vectors obtained by time-delay embedding of the data. The SL takes on values between p_ref_ (no coupling) and 1 (complete coupling).

### Data Processing

EEG data were analyzed in three frequency bands: 4–8 Hz, 8–10 Hz, and 10–13 Hz and in the second stage, i.e., observation stage (4 s–14 s). Data processing was performed in five steps. First, in the case of channels from which the data were evaluated as unexpectedly corrupted by noise, those channels were reconstructed as a linear combination of their nearest neighbours (Scan 4.4 software, Revision E, 2007). Second, EEG data were reformatted in a common average montage. Stam and de Bruin [42] have shown that montages using mastoids as a reference accentuated long-distance coupling at the cost of small-scale detail, whereas source montages displayed the reverse. Common average montages had intermediate properties [43] and such a montage was used in the present study. Third, ocular (blink) artifacts were reduced via spatial filtering. Artifacts were identified within the data source file with voltage thresholding on the VEOG channel. These were saved as events, and correlation was performed to uniformly align the events to the local signal peaks. Data segments were epoched, and outlier epochs were visually identified and rejected before the remainder was averaged. This average was used to create a SVD (Singular Value Decomposition) file that was applied as a linear derivation (spatial filtering) to the original data (Scan 4.4 software, Revision E, 2007). Fourth, the EEG trials were segmented. For the two observation conditions, only the second stage (4 s–14 s) was considered for the EEG analysis. For the two control conditions, lasting one minute, the four first seconds were discarded and the following 10 seconds were selected for analysis (4 s–14 s).

Finally, SL was computed for all the 2016 electrode pairs (stemming from the 64 electrode sites) for the second stage of each of the trials of the two observation conditions, for each subject, and frequency band. The 2016 SL values were averaged across trials for each subject, observation condition, and frequency band. For the 10 second-period of the two control conditions, the same procedure was adopted. Parameters for the computation of the synchronization likelihood were: 10 sample for the lag; 10 for the embedding dimension; 100 for the Theiler correction; 0.05 for the P_ref_; and 8 for the speed. To diminish the variability between subjects and electrode pairs, the SL value under the control condition was subtracted from the SL value under the observation condition as stated by the formula: SL_final_ = SL_observation condition_−SL_control condition_ [44], [45]. A positive SL_final_ value indicated a SL increase, whereas a negative value represented a SL decrease. The subtraction of control SL values from experimental (observation condition) values were also undertaken to remove synchronizations which occurred during the control and experimental conditions and which were not related to the task to be performed.

### Statistical Analysis

**Figure 2:**
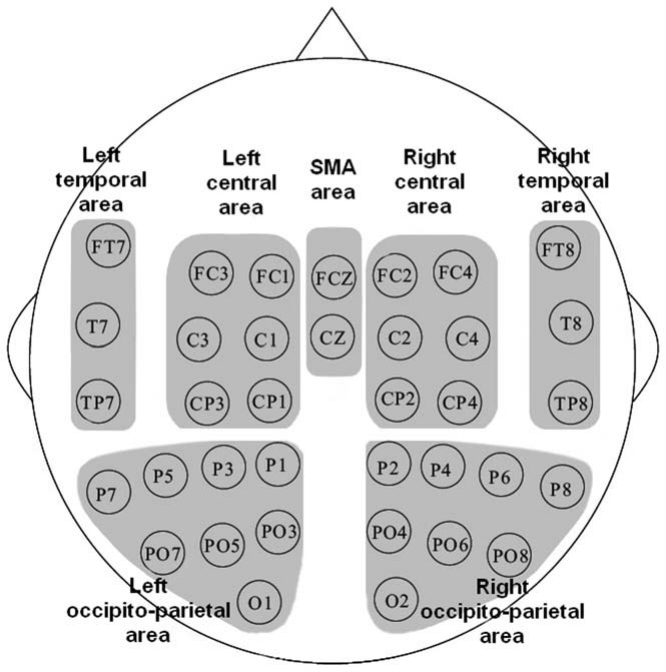
The areas of interest.

To reduce the degrees of freedom in the statistical analyses, SL_final_ from neighbouring electrode sites were averaged together to obtain one overall SL_final_ value for each of the seven following areas: right central area (FC2, FC4, C2, C4, CP2, CP4), left central area (FC3, FC1, C3, C1, CP3, CP1), SMA area (FCZ, CZ), right temporal area (FT8, T8, TP8), left temporal area (FT7, T7, TP7), right occipito-parietal area (P2, P4, P6, P8, PO4, PO6, PO8, O2), and left occipito-parietal area (P7, P5, P3, P1, PO7, PO5, PO3, O1) (see Figure 2). FC3, FC1, C3, C1, CP3, CP1 and FC2, FC4, C2, C4, CP2, CP4 were included because these sites are know to overlie approximately the lateral premotor cortex, the primary sensorimotor cortex of respectively the left and right hemispheres [44], [46] which constitute a network of cortical motor-related areas [44]. FCZ and CZ electrode sites were selected because these sites are recognized to overlie the SMA [44] which is involved in action observation [26], [27] and in the programming and planning of internally triggered behaviours [47]. FT8, T8, TP8, FT7, T7, TP7 electrode sites were taken into consideration since the superior temporal sulcus (STS), which is located in the temporal area, is perceived to play a role in the perception of biological motions [22], [25]. Occipito-parietal areas were considered since they are involved in visual perception [23], [24]. However, the reader must be aware that the potential distribution over the scalp does not precisely determine the sources which generate this distribution [48]. Then, for each of the three frequency bands, subject, and area, one SL_final_ value was obtained.

All statistical analyses were performed using Statistica 7.1. First, before completing the subtraction between SL_final_ values under the observation and control conditions, SL of the two control conditions were statistically compared. If there were any differences between both control conditions, calculation of SL_final_ would not be very informative. Pairewise Wilcoxon tests with Bonferroni correction were conducted since assumptions for the use of ANOVA or MANOVA were not satisfied. The data were not normally distributed and the hypothesis of sphericity was violated. To address the problem of multiple comparisons, significance levels for the Wilcoxon's tests were adjusted providing an alpha level of *p*<.00238 since 21 comparisons between the two control conditions were examined within each of the seven areas and for each of the three frequency bands.

Second, for each of the three frequency bands, 2 (conditions)×7 (areas) multivariate analysis of variance (MANOVAs) were completed. There was one dependent variable; condition (two levels: video motion observation, point-light motion observation) and one independent variable; area (seven levels: right central area, left central area, SMA area, right temporal area, left temporal area, right occipito-parietal area, left occipito-parietal area). Computing MANOVAs instead of repeated measures ANOVAs was chosen because in the present case, the assumption of sphericity in repeated measures ANOVA designs is violated. Since MANOVAs do not assume sphericity, this option has been selected [49]. Because three MANOVAs were computed (i.e., one MANOVA for each of the three frequency bands of interest), a correction of *p* value for multiple testing was applied. To tackle with this multiplicity problem, the False Discovery Rate (FDR) [50] was used. Planned comparisons were calculating where MANOVA results were significant. Planned comparisons were conducted between SL_final_ values under the point-light and the video observation conditions for each area. Because these planned comparisons were orthogonal, there was no need to adjust the alpha level [51]. Effect sizes (ES) for repeated measures [52] were also reported. ES allows to judge the importance of the difference size between two variables [53]–[55]. Its report with *p* values is beneficial since it allows the reader to assess the significance of the results [55]. It also enables a comparison across studies whatever the size of the samples [55]. In the present study, a positive ES indicates a higher SL_final_ value under the point-light motion observation condition compared to the SL_final_ value under the video motion observation condition. A negative ES indicates a higher SL_final_ value under the video motion observation condition. Values suggested by Cohen [53] were employed to quantify the effect (d = .20 for small effect; d = .50 for medium effect, and d = .80 for large effect). Before the MANOVA computations, the normality of the data was checked with the Kolmogorov Smirnov test. Univariate, multivariate and residual normality of the EEG data were checked.

## Results

### Behavioral Results

During the point-light motion observation and video motion observation conditions, the percentages of correct answers performed by the subjects were, respectively, 94% (SD = 4.92) and 96.33% (SD = 4.83). This difference was not statistically significant (Wilcoxon, T = 10.5, *p* = .30). The subjects also reported that the observation of movements from a video display was easier compared to that of the same movements from a point-light display (8.28 vs 6.28, Wilcoxon, T = 0.00, p = .007686).

### Synchronization Likelihood Results

Computation of Wilcoxon tests with Bonferroni correction revealed no significant SL differences between the two control conditions within each of the seven areas and for each of the three frequency bands (see Table 1).

**Table 1:** *p* values for pairewise Wilcoxon tests with Bonferroni correction for the three frequency band of interest.

Pairewise Comparisons	4–8 Hz	8–10 Hz	10–13 Hz
Right central area Point-Light Control *vs* Right central area Video Control	.575	.284	.799
Left central area Point-Light Control *vs* Left central area Video Control	.721	.114	.507
SMA area Point-Light Control *vs* SMA area Video Control	.007	.012	.575
Right temporal area Point-Light Control *vs* Right temporal area Video Control	.169	.169	.959
Left temporal area Point-Light Control *vs* Left temporal area Video Control	.169	.333	.646
Right occipito-parietal area Point-Light Control *vs* Right occipito-parietal area Video Control	.241	.445	.799
Left occipito-parietal area Point-Light Control *vs* Left occipito-parietal area Video Control	.386	.799	.799

*p* corrected value should be inferior to .00238. No significant SL differences were found between the two control conditions within each of the seven areas and for the three frequency bands.

SL_final_ values under the two observation conditions were normally distributed. *p* values superior to .05 were reported as univariate, multivariate, and residual normality was checked by Kolmogorov Smirnov test. FDR analysis [50] was conducted to consider correction of p value for multiple testing and the alpha level to demonstrate significance was *p*<.033. Three 2(conditions)×7(areas) MANOVAs were computed. Significant conditions by areas interactions were found for: (i) the 4–8 Hz band, F(6, 63) = 2.710, *p* = .021; (ii) the 8–10 Hz band, F(6, 63) = 2.562, *p* = .028. Planned comparison analyses were performed and two results were obtained.

**Figure 3:**
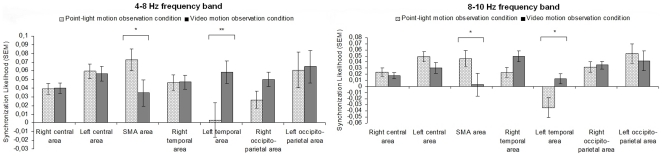
SL_final_ values for each area of interest in the 4–8 Hz and 8–10 Hz frequency bands under the point-light motion observation and video motion observation conditions. Asterisks (*) indicate statistically significant differences between the two conditions. * p<.05, ** p<.003.

First, in the 4–8 Hz frequency band, planned comparison analyses revealed significant differences for the SMA and the left temporal areas between the point-light motion observation condition and the video motion observation condition (see Figure 3). In the SMA area, the SL_final_ value under the point-light motion observation condition was higher compared to the SL_final_ value under the video motion observation condition (*p*<.05, ES = 0.41). The opposite result was observed for the left temporal area (*p*<.003, ES = −0.49) (see Figure 3).

Second, in the 8–10 Hz frequency band, planned comparison analyses also identified significant differences for the SMA and the left temporal areas between the two conditions (see Figure 3). In the SMA area, the SL_final_ value under the point-light motion observation condition was greater than the SL_final_ value under the video motion observation condition (*p*<.05, ES = 0.41). In the left temporal area, SL_final_ values were opposite in sign (*p*<.05, ES = −0.59): A SL_final_ decrease was developed under the point-light motion observation condition, whereas under the video motion observation condition a SL_final_ increase was produced (see Figure 3).

## Discussion

The aim of this study was to shed some light on the processing involved in human visual perception of biological motion. Functional connectivity in response to different visual stimuli depicting highly familiar motion was investigated. It was found that in the 4–8 Hz and 8–10 Hz frequency bands, functional connectivity within the SMA area was greater during the observation of familiar biological motions embedded in a highly unfamiliar display (i.e., point-light display) compared to the observation of familiar motions embedded in a highly familiar display (i.e., video display). The reverse was observed for the left temporal area.

The discussion is organized into three sections. The first two sections discuss the differences in functional connectivity between familiar motions presented in point-light and video displays within the SMA and (left) temporal areas in the 4–8 Hz and 8–10 Hz frequency bands. The final section considers methodological issues.

To avoid confusion throughout the course of the discussion, the reader should bear in mind that the term ‘synchronization’ has been used in the literature with varied definitions. One definition of synchronization has been the mechanism for integrating and representing information in the brain. Different, spatially separated brain regions communicate via a process where large groups of neurons fire in synchrony (e.g., [15], [56], [57]). A second meaning is that used by Pfurtscheller's research group. Synchronization is inferred from specific local band power changes. Consequently, these changes cannot be interpreted in an unambiguous way in terms of changes in coupling in the underlying network. More specifically, a decrease in relative power is termed event-related desynchronization (ERD) and an increase is defined as event-related synchronization (ERS). An ERD is a marker of an active cortical processing [58], whereas an ERS reflects an idling state [59] or even a state of cortical inhibition [60], [61].

### Functional Connectivity Differences in the 4–8 Hz Frequency Band

Consistent with our prediction, differences in functional connectivity between displays of moving humans were observed for the theta (4–8 Hz) frequency band. These findings are in line with those of Klimesch et al. [29], Sarnthein et al. [62], and Stam et al. [19]. The utilization of a local power index [29] or a global interregional synchronization index [19], [62] has shown that theta oscillations are found to be closely related to the memory encoding process. In the present study, the paradigm employed, mirrors that used in memory literature. In fact, the subjects encode information, during the observation stage, to keep it momentarily in working memory before performing a recognition task after an interval of a few seconds.

The differences in functional connectivity within the SMA are not unexpected. This region, known to participate in the processing and planning of internally triggered behaviours [47], has also been recognized as being activated during the observation of actions [26], [27], [63]–[65]. For instance, through the use of fMRI, Zentgraf et al. [27] have demonstrated the involvement of the SMA and particularly the pre-SMA when subjects observed whole-body gymnastic movements with the purpose of evaluating them subsequently. In the same vein, Schubotz and von Cramon [65] have shown that both the SMA and pre-SMA played a role during the encoding process. These areas were activated when viewing motions with the aim to carry out a perceptual assessment at a later stage. When subjects watched point-light biological motion videos of kicking and jumping jacking, Ulloa and Pineda [26] observed a decrease in mu power at CZ electrode site which is thought to express SMA activity. More recently, Mukamel et al. [64], using recordings of extracellular activity in human cells, have shown that cells within the SMA responded to observation and execution of grasping actions.

As predicted, the analysis of the 4–8 Hz frequency band showed that the SL_final_ increase was greater under the point-light motion observation than under the video motion observation condition within the SMA area (FCZ and CZ). In the present study, the subjects reported more difficulty in watching biological motions from a point-light display compared to from a video display. Consequently, viewing a motion from a minimalist display would require more mental effort compared to viewing this motion from a video display since the subjects are not accustomed to experiencing these artificial stimuli in the daily life. This suggestion adds weight to the suggestion by Onton et al. [31]: Theta is related to the level of mental effort required to complete a task. It can also be assumed that this mental effort could generate a greater cognitive demand and could be expressed by a higher functional connectivity under the point-light motion observation condition. This extra cognitive demand could also explain that behavioral results did not highlight a significant difference in the percentage of correct recognition between the two observation conditions because of the successful cognitive compensation. This interpretation is consistent with the findings of Kahana et al. [66] which have shown a theta increase with task difficulty. For instance, using intracranial EEG in epileptic subjects, Kahana et al. [66] have demonstrated during virtual maze navigation tasks that distinct theta (oscillation) episodes occurred more frequently in complex mazes than in simple ones. The reader must be aware that using ERD, a method pioneered by Pfurtscheller and Aranibar [17], Klimesch and co-workers (see [30] for a review) also revealed a relationship between theta increase and task difficulty. The increased difficulty of a task, the stronger is the magnitude of theta synchronization.

Based on the SMA result, a question arises as to whether a similar pattern in functional connectivity is not revealed within the left temporal area: SL_final_ increase was greater under the video motion observation than under the point-light motion observation condition. To discuss this finding, we focus on the results from brain imaging studies and on the fact that activity at FT7, T7, and TP7 electrode sites may indirectly reflect the activity in the STS. Michels et al. [67], Puce et al. [68], and Vaina et al. [69] suggested that the STS processes information about form and motion. Their suggestion is based on anatomical knowledge: The STS receives projections from the dorsal and ventral pathways, which handle essentially, for the former, motion information and, for the latter, colour and form information. The fact that the STS integrates form and motion information may explain the higher functional connectivity within the (left) temporal area during the observation of motion from a video display. Motions within a video display, which are laden with colour and form information, are expected to activate more strongly the STS compared to motions within a point-light display, which are devoid of colour and form information. If this explanation is taken for granted, why no significant differences between the two observation conditions are not revealed within the right temporal area. Related work by Peuskens et al. [25] can offer some support to answer this question. They showed that the right posterior STS responded strongly to human motion. Four point-light displays were compared: (i) a biological motion, (ii) a scrambled motion, (iii) a 3D rotation of a human figure, and (iv) a 3D rotation of a scrambled frame (3D cloud). The results revealed that, within the right STS, the difference between biological motion minus scrambled motion was greater than the difference between 3D rotation of a human figure minus 3D rotation of a scrambled frame. In the present study, the lack of difference in functional connectivity within the right temporal area when the subjects observed motion presented in an unfamiliar display and motion presented in a familiar display may confirm that the right STS is engaged predominantly in the treatment of motion. In other words, when video and point-light observation conditions are compared, no (SL) differences are detected since the right STS mainly respond to motion information present in the two observation conditions. Under the video observation condition, form (i.e., contextual) information are not processed.

### Functional Connectivity Differences in the 8–10 Hz Frequency Band

The differences revealed in lower alpha oscillations are not surprising since these oscillations are recognized as being involved in attentional processes [19], [30]. Recently, analyzing point-light stimuli (i.e., gathering sparse moving dots into a meaningful global form) has been recognized as a process requiring attention [5]–[10] when visual stimuli were degraded, ambiguous. Work by Chandrasekaran and co-workers [70] has even demonstrated a relationship between selective attention and the ability to recognize a point-light motion embedded in a noisy background. Observers, who exhibited greater ability to focus on relevant targets, were better in treating point-light information. Interestingly, the subjects of the study through informal reports stated that watching point-light motions was more demanding in term of attentional resources than watching video motions. They even declared that they paid more attention to information related to body parts such as upper and lower limbs under the point-light observation condition. These reports seem to imply that even in the absence of degraded, ambiguous viewing conditions, as those employed by Chandrasekaran et al. [11], Pavlova et al. [9], Safford et al. [10] or Thornthon et al. [7], attention appears to be involved in the processing of biological motions embedded in point-light displays. It can thus be suggested that watching a familiar motion within an unfamiliar display such as a point-light display, might require, additionally to a bottom-up processing, a top-down processing such as attention. Other top-down processes such as prior knowledge or/and expectations and/or thoughts of the observer agent may have intervened during the point-light motion observation but the experimental procedure in the present study does not allow us to provide answers. Additional investigation has yet to be conducted.

As expected, the SL_final_.value within the SMA was greater under the point-light motion observation condition than under the video motion observation condition. An explanation, comparable to that mentioned above, can be put forward. Interpreting point-light stimuli may have required some attentional resources. These attentional demands may have a cost and can recruit additional neuron populations. This extra recruitment can thus be expressed by higher values of functional connectivity under the point-light motion observation condition which had been perceived as a more difficult and complex condition compared to the video motion observation condition. This interpretation is not in accordance with findings observed in a study by Calmels et al. [71]. The authors did not observe any differences in functional connectivity in the 8–10 Hz frequency band between a simple observation condition and a complex observation condition at FZ and CZ electrode sites. Though functional connectivity was assessed by the same indicator in the two studies, the discordant results could be related to: (i) the different nature of the tasks (sequential finger movement *vs* whole body movement), (ii) the intentions of the observer (observation for replica *vs* observation for recognition), and (iii) the problem to distinguish, in Calmels et al. [70], the level of difficulty between the two finger movements. However, the results in the 8–10 Hz frequency band corroborate the findings of Klimesch et al. [29], Klimesch [72], and Boiten et al. [73]. These authors revealed that with increasing attentional demand, (local) power alpha power decreased meaning that the capacity of the cortex to treat information was increased.

The next point that needs to be addressed is why the functional connectivity pattern observed within the left temporal area under the point-light display is dissimilar to the one detected within the SMA area. Indeed, the pattern within the left temporal area displays a negative value under the point-light motion observation condition. This negative value expresses a synchronization decrease to below the level of the control condition. On the basis of work by Klimesch et al. [74] and Krause et al. [75], it can be speculated that the capacity limit of attentional resources were exceeded in this particular cortical area. This may have led to an inhibition which could have inhibited irrelevant information and/or blocked information related to previous trials to prevent interference during the encoding of new information. More recently, Klimesch et al. [61] argued that alpha ERS, which reflects a state of cortical inhibition, can be better expressed as a “top-down control.” They defined it as “an attentional control function that keeps processes focused on highly selective aspects of task performance by using inhibition to prevent interference from task irrelevant brain areas or processing systems” (Klimesch et al. [61], p. 69). Interestingly, the (left) temporal area, where this synchronization decrease occurs, is the area involved in the inverse circuit model presented by Gazzola and Keysers [28] which receives visual inputs and transmits them to the PPC. However, an explanation for this synchronization decrease and the area where it occurred is not yet available. To shed some light on this inhibitory mechanism, an additional experiment could be potentially conducted in which distracting information will be enhanced. If the (left) temporal area displays a more prominent decrease compared to that observed in the present study, this inhibitory suggestion will be warranted.

### Methodological Issues

Caution must be exerted when interpreting the results of this study. First, results based on EEG signals do not reflect similar aspects of cortical activity obtained by other techniques, such as fMRI and TMS. For example, it is difficult to discuss the results of the present study in the same context as findings reported in Alaerts et al. [76] since the techniques used, the areas investigated, and the motions observed, all differ: EEG *vs* TMS, all scalp areas *vs* primary motor cortex, whole body motions *vs* hand motions. Second, attention should also be paid to the experimental design. Our results are not easily comparable with the findings in studies which have taken into consideration only the point-light motion observation condition and which have discarded the video motion observation condition from the experimental procedure (e.g., [26]). Third, caution should be exercised in the comparison of EEG studies using different data analysis procedures. For example, the comparison of local power changes (e.g., ERD/ERS; [29], [30], [72], [74]) with global interregional synchronization (e.g., functional connectivity; [14], [15]) is not straightforward since these two indicators are distinct phenomena which occur simultaneously and display different spatiotemporal patterns [77]. Fourth, a lack of significant difference in EEG activity between two experimental conditions does not automatically imply equality. Activity differences could exist but EEG as a technique may be unable to detect these differences which may be related to deeper motor structures (e.g., basal ganglia or thalamus) of which activity is not present at the scalp [78]. Finally, the recruitment of the subjects could have been enlarged by including different kinds of people to gain further insight into the processing of biological motions in humans. It would have been of interest to include sedentary people observing regular movement and to compare them to the sample of the present study to check whether the results obtained in expert gymnasts could be generalized to results in the general population. Incorporating people who possess a high visual familiarity of the movements, such as judges or coaches, and comparing them to expert gymnasts who possess a high visual and motor familiarity would have allowed us to investigate the influence of motor familiarity upon the perception of motions. Besides, involving novice athletes with no visual and motor familiarity would have presented an advantage to study the impact of expertise on human visual perception.

### Conclusion

The findings of the present study indicate that the visual display depicting familiar biological motions influences functional connectivity in lower frequency bands. More specifically, functional connectivity within the SMA was greater during the observation of biological motions embedded in unfamiliar point-light displays compared to the observation of motions embedded in familiar video displays. The reverse was observed for the 4–8 Hz frequency band within the left temporal area. These results suggest that viewing a familiar motion presented in an unfamiliar display require, additionally to a bottom-up processing, a top-down processing such as attention.

## References

[1] Johansson G (1973). Visual perception of biological motion and a model for its analysis.. Percept Psychophys.

[2] Blake R, Shiffrar M (2007). Perception of human motion.. Annu Rev Psychol.

[3] Giese MA, Poggio T (2003). Neural mechanisms for the recognition of biological movements.. Nat Rev Neurosci.

[4] Mather G, Radford K, West S (1992). Low-level visual processing of biological motion.. Proc Biol Sci.

[5] Battelli L, Cavanagh P, Thornton IM (2003). Perception of biological motion in parietal patients.. Neuropsychologia.

[6] Cavanagh P, Labianca AT, Thornton IM (2001). Attention-based visual routines: sprites.. Cognition.

[7] Thornton IM, Rensink RA, Shiffrar M (2002). Active versus passive processing of biological motion.. Perception.

[8] Parasuraman R, de Visser E, Clarke E, McGarry WR, Hussey E (2009). Detecting threat-related intentional actions of others: effects of image quality, response mode, and target cuing on vigilance.. J Exp Psychol Appl.

[9] Pavlova M, Birbaumer N, Sokolov A (2006). Attentional modulation of cortical neuromagnetic gamma response to biological movement.. Cereb Cortex.

[10] Safford AS, Hussey EA, Parasuraman R, Thompson JC (2010). Object-based attentional modulation of biological motion processing: spatiotemporal dynamics using functional magnetic resonance imaging and electroencephalography.. J Neurosci.

[11] Chandrasekaran C, Thornton IM, Bülthoff HH (2005). Selective attention to biological motion (Report No 139).

[12] Beauchamp MS, Lee KE, Haxby JV, Martin A (2003). FMRI responses to video and point-light displays of moving humans and manipulable objects.. J Cogn Neurosci.

[13] Grossman ED, Blake R (2002). Brain Areas Active during Visual Perception of Biological Motion.. Neuron.

[14] Montez T, Linkenkaer-Hansen K, van Dijk BW, Stam CJ (2006). Synchronization likelihood with explicit time-frequency priors.. Neuroimage.

[15] Stam CJ, Van Dijk BW (2002). Synchronization likelihood: an unbiased measure of generalized synchronization in multivariate data sets.. Physica D.

[16] Pfurtscheller G (1988). Mapping of event-related desynchronization and type of derivation.. Electroencephalogr Clin Neurophysiol.

[17] Pfurtscheller G, Aranibar A (1977). Event-related cortical desynchronization detected by power measurements of scalp EEG.. Electroencephalogr Clin Neurophysiol.

[18] Salmelin R, Hari R (1994). Spatiotemporal characteristics of sensorimotor neuromagnetic rhythms related to thumb movement.. Neuroscience.

[19] Stam CJ, van Cappellen van Walsum AM, Micheloyannis S (2002). Variability of EEG synchronization during a working memory task in healthy subjects.. Int J Psychophysiol.

[20] Fingelkurts AA, Fingelkurts AA, Kahkonen S (2005). Functional connectivity in the brain–is it an elusive concept?. Neurosci Biobehav Rev.

[21] Friston KJ (2000). The labile brain. I. Neuronal transients and nonlinear coupling.. Philos Trans R Soc Lond B Biol Sci.

[22] Allison T, Puce A, McCarthy G (2000). Social perception from visual cues: role of the STS region.. Trends Cogn Sci.

[23] Haxby JV, Grady CL, Horwitz B, Ungerleider LG, Mishkin M (1991). Dissociation of object and spatial visual processing pathways in human extrastriate cortex.. Proc Natl Acad Sci U S A.

[24] Mishkin M, Ungerleider LG, Macko KA (1983). Object vision and spatial vision: two cortical pathways.. Trends Neurosci.

[25] Peuskens H, Vanrie J, Verfaillie K, Orban GA (2005). Specificity of regions processing biological motion.. Eur J Neurosci.

[26] Ulloa ER, Pineda JA (2007). Recognition of point-light biological motion: mu rhythms and mirror neuron activity.. Behav Brain Res.

[27] Zentgraf K, Stark R, Reiser M, Kunzell S, Schienle A (2005). Differential activation of pre-SMA and SMA proper during action observation: effects of instructions.. Neuroimage.

[28] Gazzola V, Keysers C (2009). The observation and execution of actions share motor and somatosensory voxels in all tested subjects: single-subject analyses of unsmoothed fMRI data.. Cereb Cortex.

[29] Klimesch W, Schimke H, Schwaiger J (1994). Episodic and semantic memory: an analysis in the EEG theta and alpha band.. Electroencephalogr Clin Neurophysiol.

[30] Klimesch W (1999). EEG alpha and theta oscillations reflect cognitive and memory performance: a review and analysis.. Brain Res Brain Res Rev.

[31] Onton J, Delorme A, Makeig S (2005). Frontal midline EEG dynamics during working memory.. Neuroimage.

[32] Calvo-Merino B, Glaser DE, Grezes J, Passingham RE, Haggard P (2005). Action observation and acquired motor skills: an FMRI study with expert dancers.. Cereb Cortex.

[33] Calvo-Merino B, Grezes J, Glaser DE, Passingham RE, Haggard P (2006). Seeing or doing? Influence of visual and motor familiarity in action observation.. Curr Biol.

[34] Oldfield RC (1971). The assessment and analysis of handedness: the Edinburgh inventory.. Neuropsychologia.

[35] Grafton ST, Arbib MA, Fadiga L, Rizzolatti G (1996). Localization of grasp representations in humans by positron emission tomography. 2. Observation compared with imagination.. Exp Brain Res.

[36] Jonas M, Siebner HR, Biermann-Ruben K, Kessler K, Baumer T (2007). Do simple intransitive finger movements consistently activate frontoparietal mirror neuron areas in humans?. Neuroimage.

[37] Urgesi C, Candidi M, Fabbro F, Romani M, Aglioti SM (2006). Motor facilitation during action observation: topographic mapping of the target muscle and influence of the onlooker's posture.. Eur J Neurosci.

[38] Grossman E, Donnelly M, Price R, Pickens D, Morgan V (2000). Brain areas involved in perception of biological motion.. J Cogn Neurosci.

[39] Saygin AP, Wilson SM, Hagler DJ, Bates E, Sereno MI (2004). Point-light biological motion perception activates human premotor cortex.. J Neurosci.

[40] Nuwer MR, Lehmann D, da Silva FL, Matsuoka S, Sutherling W (1999). IFCN guidelines for topographic and frequency analysis of EEGs and EPs.The International Federation of Clinical Neurophysiology.. Electroencephalogr Clin Neurophysiol.

[41] Calmels C, Hars M, Jarry G, Stam CJ (2010). Non-linear EEG synchronization during observation: effects of instructions and expertise.. Psychophysiology.

[42] Stam CJ, de Bruin EA (2004). Scale-free dynamics of global functional connectivity in the human brain.. Hum Brain Mapp.

[43] Nunez PL, Srinivasan R, Westdorp AF, Wijesinghe RS, Tucker DM (1997). EEG coherency. I: Statistics, reference electrode, volume conduction, Laplacians, cortical imaging, and interpretation at multiple scales.. Electroencephalogr Clin Neurophysiol.

[44] Gerloff C, Richard J, Hadley J, Schulman AE, Honda M (1998). Functional coupling and regional activation of human cortical motor areas during simple, internally paced and externally paced finger movements.. Brain.

[45] Manganotti P, Gerloff C, Toro C, Katsuta H, Sadato N (1998). Task-related coherence and task-related spectral power changes during sequential finger movements.. Electroencephalogr Clin Neurophysiol.

[46] Babiloni C, Del Percio C, Rossini PM, Marzano N, Iacoboni M (2009). Judgment of actions in experts: a high-resolution EEG study in elite athletes.. Neuroimage.

[47] Passingham RE (1996). Attention to action.. Philos Trans R Soc Lond B Biol Sci.

[48] Lagerlund TD, Worrell GA, Niedermeyer E, Lopes da Silva F (2005). EEG source localization (model-dependent and model- independent methods).. Electroencephalography : basic principles, clinical applications, and related fields.

[49] O'Brien RG, Kaiser MK (1985). MANOVA method for analyzing repeated measures designs: an extensive primer.. Psychol Bull.

[50] Benjamini Y, Hochberg Y (1995). Controlling the false discovery rate: a practical and powerful approach to multiple testing.. J Roy Stat Soc B.

[51] Sokal RR, Rohlf FJ (1995). Biometry: the principles and practice of statistics in biological research.

[52] Long BC, van Stavel R (1995). Effects of exercise training on anxiety: A meta-analysis.. J Appl Sport Psychol.

[53] Cohen J (1988). Statistical power analysis for the behavioral sciences (2nd ed.).

[54] Garamszegi LZ (2006). Comparing effect sizes across variables: generalization without the need for Bonferroni correction.. Behav Ecol.

[55] Nakagawa S (2004). A farewell to Bonferroni: the problems of low statistical power and publication bias.. Behav Ecol.

[56] Varela F, Lachaux JP, Rodriguez E, Martinerie J (2001). The brainweb: phase synchronization and large-scale integration.. Nat Rev Neurosci.

[57] Fries P (2005). A mechanism for cognitive dynamics: neuronal communication through neuronal coherence.. Trends Cogn Sci.

[58] Pfurtscheller G, Lopes da Silva FH (1999). Event-related EEG/MEG synchronization and desynchronization: basic principles.. Clin Neurophysiol.

[59] Pfurtscheller G, Stancak A, Neuper C (1996). Event-related synchronization (ERS) in the alpha band–an electrophysiological correlate of cortical idling: a review.. Int J Psychophysiol.

[60] Klimesch W (1996). Memory processes, brain oscillations and EEG synchronization.. Int J Psychophysiol.

[61] Klimesch W, Sauseng P, Hanslmayr S (2007). EEG alpha oscillations: the inhibition-timing hypothesis.. Brain Res Rev.

[62] Sarnthein J, Petsche H, Rappelsberger P, Shaw GL, von Stein A (1998). Synchronization between prefrontal and posterior association cortex during human working memory.. Proc Natl Acad Sci U S A.

[63] Grezes J, Decety J (2001). Functional anatomy of execution, mental simulation, observation, and verb generation of actions: a meta-analysis.. Hum Brain Mapp.

[64] Mukamel R, Ekstrom AD, Kaplan J, Iacoboni M, Fried I (2010). Single-Neuron Responses in Humans during Execution and Observation of Actions.. Curr Biol.

[65] Schubotz RI, von Cramon DY (2001). Interval and ordinal properties of sequences are associated with distinct premotor areas.. Cereb Cortex.

[66] Kahana MJ, Sekuler R, Caplan JB, Kirschen M, Madsen JR (1999). Human theta oscillations exhibit task dependence during virtual maze navigation.. Nature.

[67] Michels L, Lappe M, Vaina LM (2005). Visual areas involved in the perception of human movement from dynamic form analysis.. Neuroreport.

[68] Puce A, Syngeniotis A, Thompson JC, Abbott DF, Wheaton KJ (2003). The human temporal lobe integrates facial form and motion: evidence from fMRI and ERP studies.. Neuroimage.

[69] Vaina LM, Solomon J, Chowdhury S, Sinha P, Belliveau JW (2001). Functional neuroanatomy of biological motion perception in humans.. Proc Natl Acad Sci U S A.

[70] Chandrasekaran C, Turner L, Bülthoff HH, Thornton IM (2010). Attentional networks and biological motion.. Psihologija.

[71] Calmels C, Hars M, Holmes P, Jarry G, Stam CJ (2008). Non-linear EEG synchronization during observation and execution of simple and complex sequential finger movements.. Exp Brain Res.

[72] Klimesch W (1997). EEG-alpha rhythms and memory processes.. Int J Psychophysiol.

[73] Boiten F, Sergeant J, Geuze R (1992). Event-related desynchronization: the effects of energetic and computational demands.. Electroencephalogr Clin Neurophysiol.

[74] Klimesch W, Doppelmayr M, Schwaiger J, Auinger P, Winkler T (1999). ‘Paradoxical’ alpha synchronization in a memory task.. Brain Res Cogn Brain Res.

[75] Krause CM, Sillanmaki L, Koivisto M, Saarela C, Haggqvist A (2000). The effects of memory load on event-related EEG desynchronization and synchronization.. Clin Neurophysiol.

[76] Alaerts K, Van Aggelpoel T, Swinnen SP, Wenderoth N (2009). Observing shadow motions: resonant activity within the observer's motor system?. Neurosci Lett.

[77] Calmels C, Jarry G, Stam CJ (2009). Changes in local and distant EEG activities before, during and after the observation and execution of sequential finger movements.. Neurophysiol Clin.

[78] Kahana MJ, Seelig D, Madsen JR (2001). Theta returns.. Curr Opin Neurobiol.

